# New microRNA-based therapies reveal common targets in paediatric medulloblastoma and adult glioblastoma

**DOI:** 10.1038/s41598-025-05517-9

**Published:** 2025-07-02

**Authors:** Denis Mustafov, Laura Thomas, Shoib S. Siddiqui, George I. Lambrou, Maria Braoudaki

**Affiliations:** 1 School of Health, Medicine and Life Sciences, University of Hertfordshire, Hatfield, AL10 9AB UK; 2 College of Health, Medicine and Life Sciences, Brunel University London, Uxbridge, UB8 3PH UK; 3 Choremeio Research Laboratory, First Department of Pediatrics, School of Medicine, National and Kapodistrian University of Athens, Thivon & Levadeias 8, Goudi, 11527 Athens, Greece; 4 University Research Institute of Maternal and Child Health & Precision Medicine, National and Kapodistrian University of Athens, Thivon & Levadeias 8, 11527 Athens, Greece

**Keywords:** Cancer, Molecular biology, Neuroscience

## Abstract

Medulloblastoma (MB), the most prevalent brain malignancy in children, presents significant challenges in paediatric oncology due to its aggressiveness and potential for relapse. Tailored treatments are crucial to mitigate treatment-related toxicities and long-term side effects on developing brains. Our study aimed to identify therapeutic targets for paediatric MB and explore common miRNA biosignatures with glioblastoma (GB), the most aggressive adult brain tumour. High-throughput small-RNA sequencing identified miR-206 and miR-383 as highly downregulated in MB samples, suggesting their tumour suppressor properties. Bioinformatics analysis identified *CORO1C* and *SV2B* as their targets. RT-qPCR, western blotting, and immunohistochemistry confirmed their overexpression in MB and GB. Elevated CORO1C expression was also found in adult MB and GB tissue samples. The role of both miRNAs on their target genes was validated through in vitro functional assays. Our study uncovers the potential role of miR-206/*CORO1C* and miR-383/*SV2B* axes as innovative therapeutic targets for combating aggressive paediatric and adult brain malignancies.

## Introduction

Medulloblastoma (MB) is the most common paediatric brain tumour, representing 6.4% of all central nervous system (CNS) tumours in children and adolescents^1^. MB originates from neural precursor cells in specific regions of the rhombic lip, with their maturation occurring in the cerebellum or brainstem during embryonic development^2^. MBs are currently classified as World Health Organisation (WHO) grade 4 embryonal tumours and are divided into four molecular subgroups^3^. Our current understanding of MB is largely based on molecular categorisation, identifying them as wingless-related integration site (WNT)-activated, sonic hedgehog (SHH)-activated, group 3, or group 4 MBs^4^. Nevertheless, the current primary treatment for these tumours includes maximal safe surgical resection, adjuvant multi-agent chemotherapy, and, for patients other than infants, risk-adapted craniospinal irradiation^5^. However, this comprehensive management approach comes with several short-term and long-term adverse effects that can impact the patients’ quality of life. Despite aggressive treatment, disease recurrence remains a significant concern, and the development of treatment resistance continues to be a persistent challenge^6^.

While MB and GB differ in patient demographics, anatomical origin, and clinical progression, they share several key molecular features that suggest overlapping mechanisms of tumorigenesis. Recent studies have shown that both tumours exhibit dysregulation of core signalling pathways such as PI3K/AKT/mTOR^7,8^, WNT^9,10^, and Notch^11,12^. Furthermore, overlapping miRNA signatures, such as miR-21, the let-7 family, and the miR-17–92 cluster, highlight shared post-transcriptional regulatory mechanisms that may contribute to tumour maintenance and therapy resistance^13,14^. These molecular similarities provide a rationale for comparative analyses and inform a broader understanding of CNS tumour biology across different age groups.

GB is the most frequent form of primary brain malignancy in adults with a median age at diagnosis of 65 years and an overall survival rate between 6 to 17 months^15^. It is about 1.6 times more common in males than in females, although the reason for this discrepancy remains unclear^16^. In England, over three decades (1995–2017), approximately one-third of all diagnosed primary brain tumours were classified as GBs^17^. Diagnosing adult gliomas involves a multistep approach, starting with evaluating symptoms and medical history, followed by neurological examinations and imaging techniques like magnetic resonance imaging (MRI). Biopsies or surgical resections may be needed to confirm the type and grade of the tumour^18^. Treatment options vary based on the tumour characteristics and patient health but typically involve surgery to remove as much of the tumour as possible. Radiation and chemotherapy are often utilised to target remaining or inoperable tumours^19^. However, GB therapy faces significant challenges, including limited effectiveness and severe side effects that impact patients’ quality of life. Previous research has identified at least four major GB cell states: mesenchymal (MES), oligodendrocyte precursor-like (OPC), astrocyte-like (AC), and neural progenitor-like (NPC). These reflect the tumour’s cellular plasticity and contribute to its high degree of intratumoural heterogeneity, presenting a major barrier to effective, uniform treatment responses^20^. Despite extensive research, there is currently no cure for GB, and treatment options remain palliative rather than curative^21^.

MicroRNAs (miRNAs) hold significant potential for the treatment of MB and GB due to their ability to regulate gene expression post-transcriptionally^22^. miRNAs are small, non-coding, single-stranded RNA molecules, about 22 nucleotides long, found in eukaryotic organisms^23^. Several miRNAs exhibit dysregulated expression patterns in MB and GB caused by errors in their multistep biogenesis process and genomic rearrangements like chromosomal translocations, insertions, and deletions involving miRNA genes^24^. These changes in miRNA expression significantly impact critical MB and GB hallmarks such as proliferation, immune evasion, resistance to cell death, metastasis, and the development of chemo-resistance by regulating their target genes^25^. For MB and GB treatment, miRNAs can be utilised to target the molecular pathways involved in tumour growth and progression. Among these, miR-206 and miR-383 have emerged as promising tumour suppressors with therapeutic relevance in both malignancies. In MB, miR-206 was previously found significantly downregulated across all molecular subgroups, with low expression linked to aggressive features such as anaplastic histology, suggesting its role as a diagnostic and prognostic biomarker^26^. However, the precise mechanisms underlying miR-206 downregulation in MB remain poorly understood. miR-383 similarly functioned as a tumour suppressor in MB by targeting oncogenes such as forkhead box M1 (*FOXM1*), known to promote proliferation and metastasis^27^, although the broader regulatory network through which miR-383 exerts its effects in MB has yet to be fully elucidated. In GB, although miR-206 has been less studied, existing data demonstrated a consistent reduction in expression in tumour tissue compared to normal brain, with downregulation associated with higher grade and poorer survival^28^. The pathways contributing to miR-206 repression in GB, however, are not well characterised. Likewise, miR-383 was observed to be downregulated in glioma and inversely correlated with tumour grade^29^, but its specific targets and mechanisms of action in GB remain to be clearly defined. By restoring the normal expression levels of dysregulated miRNAs or inhibiting the function of oncogenic miRNAs (oncomiRs), it may be possible to develop shared, more effective and less toxic therapeutic strategies for both MB and GB^30,31^.

Our investigation aimed to identify therapeutic targets for paediatric MB and explore common miRNA signatures with adult GB. Extensive high-throughput and in silico analyses using patient samples and cell lines suggested that miR-206 and miR-383 exhibit tumour-suppressive roles in both malignancies, potentially serving as targeted therapeutic interventions for both MB and GB. Furthermore, we found that restoring normal levels of these miRNAs suppressed their target oncogenes, Coronin 1C (*CORO1C*) and synaptic vesicle glycoprotein 2B (*SV2B*), resulting in decreased viability and proliferation of MB and GB cells.

## Results

### miR-206 and miR-383 are downregulated in MB and associated with cancer-related pathways

**Fig. 1:**
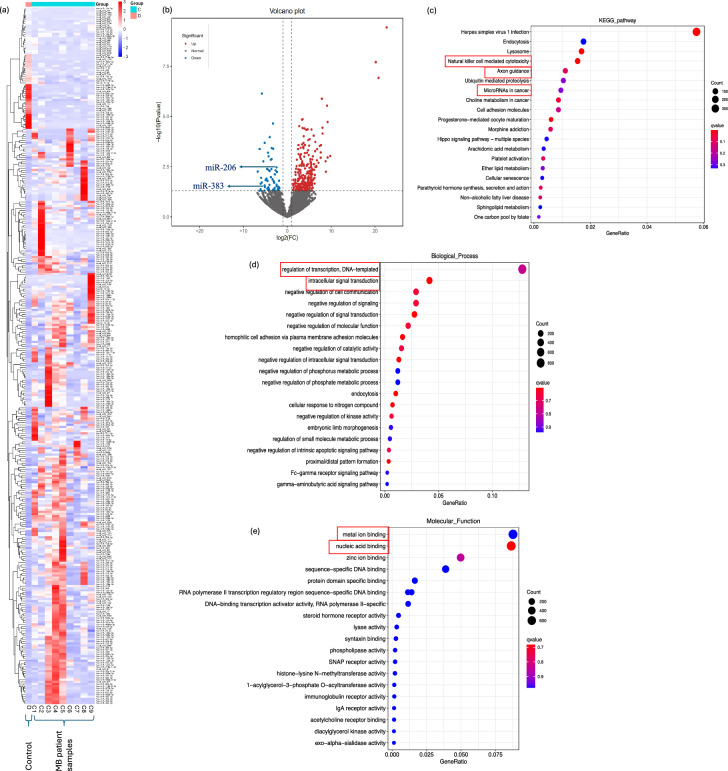
Comprehensive sRNA-seq analysis of MB tissue microRNA profiles. (**a**) Heatmap of dysregulated miRNAs (control group D (D): single paediatric autopsy brain without pathology, n = 1; MB group C (C1-C9): n = 9. (**b**) Volcano plot of miRNA expression revealed 328 dysregulated miRNAs: 57 downregulated and 271 upregulated. miR-206 and miR-383 showed significant downregulation in all MB samples (p < 0.04 and p < 0.01, respectively). (**c**) KEGG^32^ pathway analysis revealed that dysregulated miRNAs were primarily associated with axon guidance, natural killer cell mediated cytotoxicity, and pathways in cancer (red boxes). (**d**) Biological process analysis showed that most dysregulated miRNAs were involved in processes such as the regulation of transcription and intracellular signal transduction (red boxes). (**e**) Molecular function analysis of dysregulated miRNA showed that they took part in metal ion binding and nucleic acid binding (red boxes). For sRNA-seq analyses, p-values were adjusted using the Benjamini–Hochberg method to control the false discovery rate. miRNAs with an adjusted p-value < 0.05 were considered differentially expressed. A q-value < 0.005 and |log₂(fold change)|≥ 1 were used as thresholds to define significant differential expression, capturing miRNAs with meaningful and potentially biologically relevant changes, even below the more stringent fold change cutoff of > 2.

Our small RNA sequencing (RNA-seq) analysis identified significant dysregulation of miRNAs in MB tissue compared to controls. The heatmap in Fig. 1a highlights a distinct expression pattern, with clear segregation between control (D) and MB (C1-C9) samples, indicating widespread miRNA dysregulation in MB tissues. Further quantification of miRNA dysregulation is illustrated on the volcano plot in Fig. 1b. The results revealed a total of 328 miRNAs with altered expression levels. Of these, 57 miRNAs were significantly downregulated, while 271 miRNAs were upregulated in MB tissues. miR-206 and miR-383 were amongst the most significantly downregulated miRNAs across all MB samples. Pathway analysis using the Kyoto Encyclopaedia of Genes and Genomes (KEGG)^32^ highlighted that the dysregulated miRNAs were predominantly associated with critical cellular pathways (Fig. 1c). Notably, the dysregulated miRNAs were linked to axon guidance, natural killer cell-mediated cytotoxicity, and various cancer-related pathways. Biological process analysis revealed that most dysregulated miRNAs are implicated in key regulatory mechanisms, such as the regulation of transcription and intracellular signal transduction (Fig. 1d). Molecular function analysis indicated that the identified dysregulated miRNAs are involved in essential cellular functions, such as metal ion binding and nucleic acid binding activities (Fig. 1e).

### *CORO1C* and *SV2B* are direct targets of miR-206 and miR-383, respectively, via 3′UTR binding

**Fig. 2:**
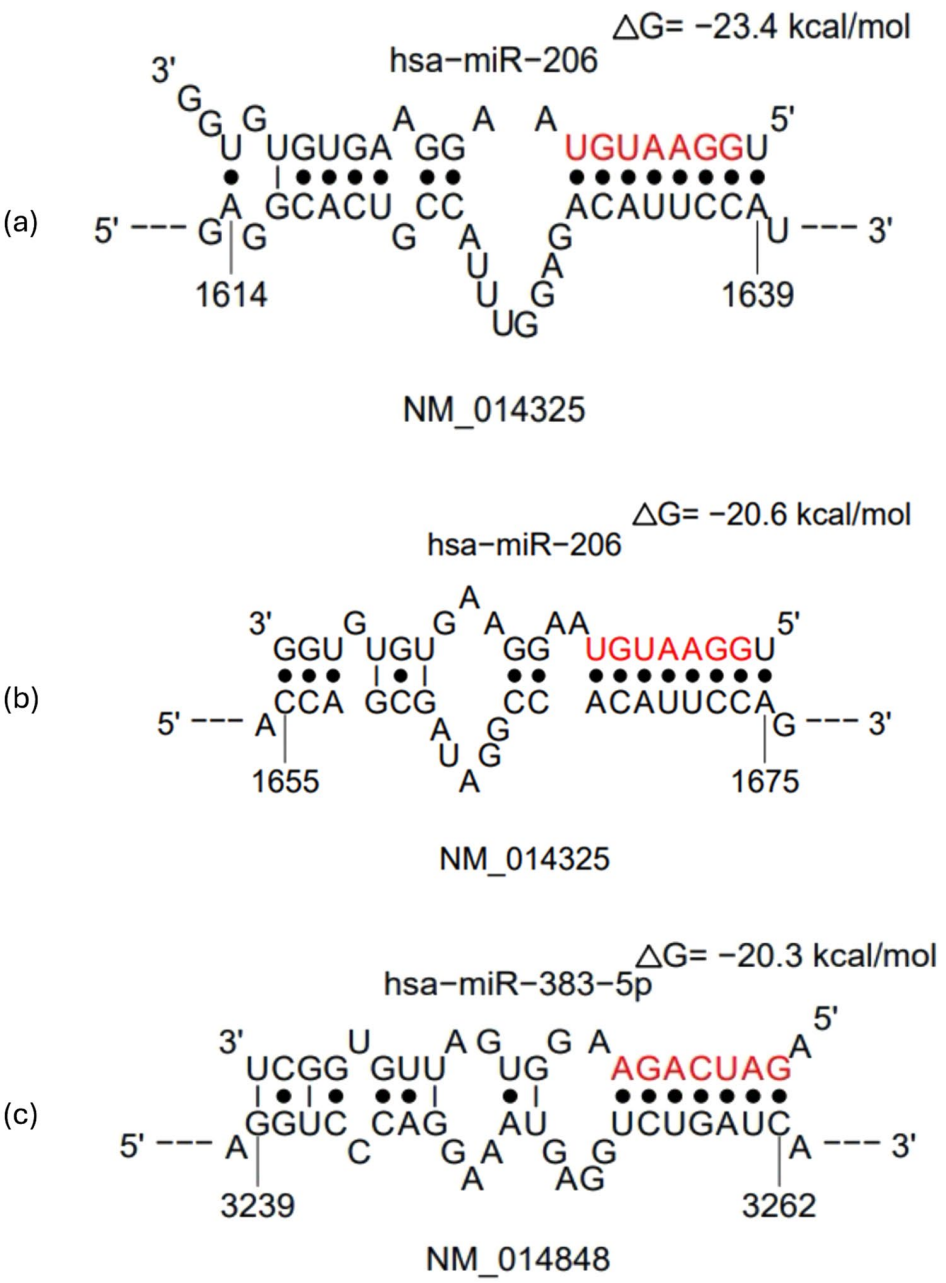
Predictive Sfold analysis illustrating the binding sites between miR-206 and *CORO1C* and miR-383-5p and *SV2B.* (**a**) 3′UTR of *CORO1C* and miR-206 binding at site position 1614–1639, ΔG hybrid =  − 23.4 kcal/mol. (**b**) 3′UTR of *CORO1C* and miR-206 binding at site position 1655–1675, ΔG hybrid =  − 20.6 kcal/mol. (**c**) 3′UTR of *SV2B* and miR-383-5p binding at site position 3239–3263, ΔG hybrid =  − 20.3 kcal/mol.

The miRNA:messenger RNA (mRNA) interaction analysis identified that miR-206 was directly targeting the *CORO1C* gene. This interaction was validated across six miRNA:mRNA interactome databases (Table 1). Furthermore, miR-383 was observed to directly target the *SV2B* gene. The interaction between them was confirmed in five miRNA:mRNA databases (Table 1). Further Sfold analysis illustrated in Fig. 2a showed the interaction between miR-206 and the 3′ untranslated region (3′UTR) of *CORO1C* at site position 1614–1639. The predicted hybridisation free energy (ΔG hybrid) for this interaction was − 23.4 kcal/mol. Figure 2b illustrated a second binding site position between miR-206 and *CORO1C* (site position 1655–1675). The predicted ΔG hybrid for this binding was − 20.6 kcal/mol. Figure 2c showed the binding interaction between miR-383-5p and the 3′UTR of *SV2B*. The analysis identified a binding site at position 3239–3263. The predicted ΔG hybrid for this interaction was − 20.3 kcal/mol.

**Table 1:** miRSystem analysis of miRNA:mRNA interactions depicted that miR-206 and miR-383 were targeting the *CORO1C* and *SV2B* genes, respectively. The interactions were validated across seven publicly available miRNA:mRNA interactome databases.

miRNA	Total hit	Validation	DIANA	MIRANDA	Mirbridge	PICTAR	PITA	RNA22	Targetscan
hsa-miR-206	6	*CORO1C*	✓		✓	✓	✓	✓	✓
hsa-miR-383-5p	5	*SV2B*	✓	✓	✓		✓	✓

### MB exhibits upregulation of *CORO1C* and *SV2B*

**Fig. 3:**
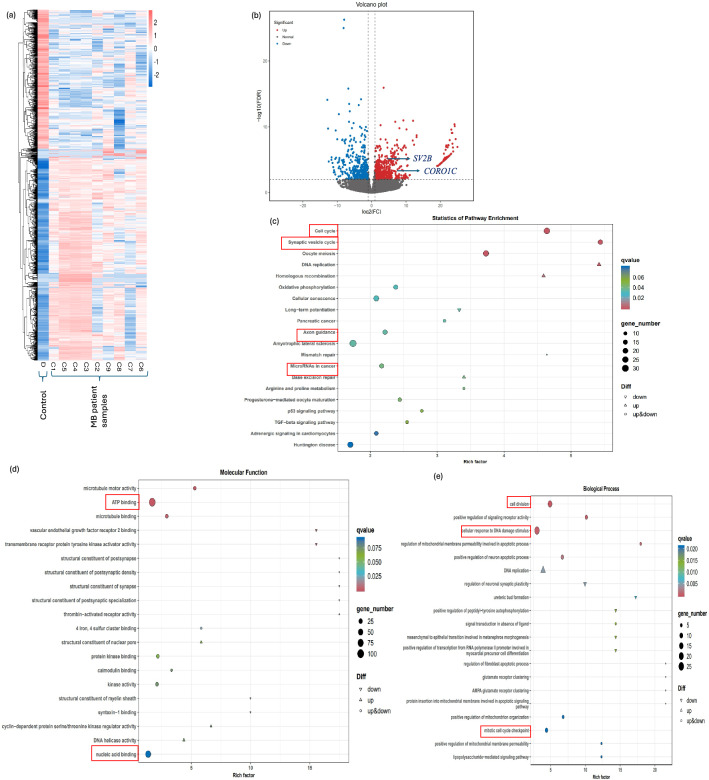
Comprehensive transcriptomics analysis of MB tissue profiles. (**a**) Heatmap of dysregulated genes (control group D (D); MB group C (C1-C9). (**b**) Volcano plot of gene expression revealed 1013 dysregulated genes: 415 downregulated and 598 upregulated. *CORO1C* and *SV2B* were identified as upregulated in the patient sample cohort. (**c**) Pathway enrichment analysis revealed that DEG were primarily associated with the regulation of the cell cycle, synaptic vesicle cycle, axon guidance, and miRNAs in cancer (red boxes). (**d**) The molecular function analysis showed that the vast majority of DEG were involved in processes such as ATP binding and nucleic acid binding (red boxes). (**e**) Biological process analysis showed that most dysregulated genes were involved cell division, cellular response to DNA damage, and mitotic cell cycle checkpoints (red boxes). Transcriptomic and sRNA-seq data were generated from the same patient cohort (n = 9). For transcriptomics analyses, p-values were adjusted using the Benjamini–Hochberg method to control the false discovery rate. Genes with an adjusted p-value < 0.05 were considered differentially expressed. A q-value < 0.005 and |log₂(fold change)|≥ 1 were used as thresholds to define significant differential expression, capturing genes with meaningful and potentially biologically relevant changes, even below the more stringent fold change cutoff of > 2.

The comprehensive transcriptomics analysis of MB tissues revealed significant dysregulation in gene expression patterns when compared to control tissues. The heatmap illustrated in Fig. 3a exhibited distinct gene expression profiles between the MB group (C1-C9) and the control group (D), indicating substantial differences in transcriptomic landscapes between normal and cancerous tissues. The volcano plot analysis (Fig. 3b) identified a total of 1013 genes with significantly altered expression levels in MB tissues. Of these, 415 genes were downregulated, while 598 genes were upregulated. The *CORO1C* and *SV2B* genes was amongst the upregulated genes in MB samples. The pathway enrichment analysis of the differentially expressed genes (DEG) revealed their association with several biological pathways, including the cell cycle, synaptic vesicle cycle, axon guidance, and miRNAs in cancer (Fig. 3c). Further analysis into the molecular functions of the DEG showed a significant involvement in ATP binding and nucleic acid binding processes (Fig. 3d). Biological process analysis highlighted that the dysregulated genes were predominantly associated with cell division, cellular response to DNA damage, and mitotic cell cycle checkpoints (Fig. 3e).

### Downregulation of miR-206 and miR-383 and increased *CORO1C* and *SV2B* expression is detected MB and GB tumour cells

**Fig. 4:**
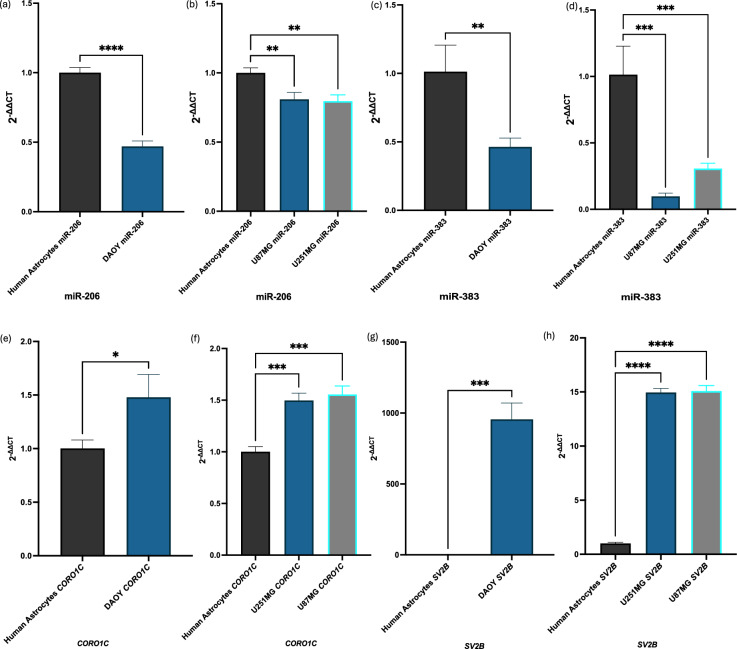
RT-qPCR validations within MB and GB cell lines. (**a**) miR-206 was significantly downregulated in MB cells in comparison to normal human astrocytes (p < 0.0001). (**b**) miR-383 was significantly downregulated in MB cells in comparison to normal human astrocytes (p < 0.0096). (**c**) *CORO1C* was significantly upregulated in MB cells in comparison to normal human astrocytes (p < 0.0218). (**d**) *SV2B* was significantly upregulated in MB cells in comparison to normal human astrocytes (p < 0.0001). (**e**) miR-206 was significantly downregulated in U87MG and U251MG GB cells in comparison to normal human astrocytes (U87MG, p < 0.0033; U251MG, p < 0.0024). (**f**) miR-383 was significantly downregulated in GB cells in comparison to normal human astrocytes (U87MG, p < 0.0002; U251MG p < 0.0009). (**g**) *CORO1C* was significantly upregulated in GB cells in comparison to normal human astrocytes (U251MG, p < 0.0002; U87MG p < 0.0001). (**h**) *SV2B* was significantly upregulated in GB cells in comparison to normal human astrocytes (U251MG, p < 0.0001; U87MG p < 0.0001). Results are based on three independent experiments, n = 3. Statistical analyses were conducted using ordinary one-way ANOVA, followed by Dunnett’s multiple comparisons test. When only two groups were compared, unpaired t-tests were used to determine significant differences in expression. Error bars represent the standard deviation (SD) of the mean.* ns* non-significant, *p < 0.05, **p < 0.01, ***p < 0.001, ****p < 0.0001.

miR-206 was found to be significantly downregulated in MB cells compared to HA-bs cells (Fig. 4a). Similarly, miR-383 exhibited a significant decrease in expression in MB cells relative to normal human astrocytes (Fig. 4b). *CORO1C* was significantly upregulated in MB cells compared to HA-bs cells (Fig. 4c). *SV2B* showed a significant increase in expression in MB cells compared to normal human astrocytes (Fig. 4d). RT-qPCR also depicted that miR-206 was significantly downregulated in U251MG and U87MG GB cells compared to HA-bs cells (Fig. 4e). The expression levels of miR-383 were significantly decreased in both GB cell lines when compared to normal human astrocytes (Fig. 4f). The *CORO1C* gene was significantly upregulated in GB cells compared to HA-bs cells (Fig. 4g). *SV2B* also showed a significant increase in expression in GB cells compared to normal human astrocytes (Fig. 4h). Survival analysis using the R2 Genomics Analysis and Visualization Platform further confirmed the clinical relevance of these targets in MB and GB. High expression of *SV2B* was significantly associated with poorer overall survival in MB patients (raw *p* = 5.18e-05; Bonferroni-adjusted *p* = 0.031), consistent with its elevated expression in MB cells (see Supplementary Fig. 2a online). Likewise, *CORO1C* expression was significantly linked to reduced survival in a subset of MB patients (raw *p* = 5.72e-05; Bonferroni-adjusted *p* = 0.034), suggesting a potential oncogenic role (see Supplementary Fig. 2b online). In GB, GEPIA-based survival analysis also revealed a significant association between high *SV2B* expression and worse overall survival (HR = 1.6, *p* = 0.015) (see Supplementary Fig. 2c online), while *CORO1C* showed a non-significant trend toward improved survival (HR = 0.72, *p* = 0.068) (see Supplementary Fig. 2d online).

### *CORO1C* and *SV2B* show distinct localisation and elevated protein expression in MB and GB cell lines

**Fig. 5:**
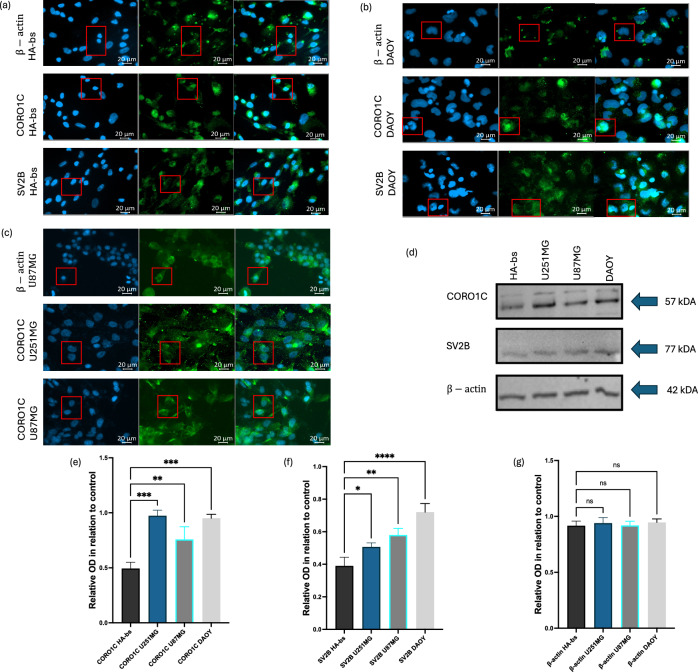
Immunofluorescence and western blot analysis of CORO1C and SV2B within HA-bs and MB cells, alongside CORO1C IF analysis within GB cells. (**a**) IF staining revealed that CORO1C, SV2B, and β-actin exhibited uniform staining across normal human astrocytes, with CORO1C showing nuclear localisation and SV2B showing cytoplasmic localisation. (**b**) IF staining within DAOY MB cells showed an increased fluorescence of SV2B in MB cells when compared to HA-bs cells, alongside a nucellar localisation of CORO1C similar to the one observed in HA-bs cells. (**c**) IF staining within GB cells revealed that there was increased fluorescence of CORO1C within both, U251MG and U87MG cells in comparison to HA-bs cells. (**d**) Chemiluminescent images showing the expression of β-actin, CORO1C, and SV2B within HA-bs, U251MG, U87MG, and DAOY cells. (**e**) CORO1C protein expression was significantly upregulated within all GB and MB cell lines in comparison to normal HA-bs cells (U251MG, p < 0.0001; U87MG, p < 0.003; DAOY, p < 0.0002). (**f**) SV2B protein expression was significantly upregulated within U251MG, U87MG, and DAOY cells in comparison to HA-bs cells (p < 0.037, p < 0.003, p < 0.0001, respectively). (**g**) β-actin protein expression revealed no significant difference between the normal astrocytes and GB and MB cells. Results are based on three independent experiments, n = 3. All statistical analyses were conducted using ordinary one-way ANOVA, followed by Dunnett’s multiple comparisons test to determine the significant differences in expression between normal human astrocytes, GB and MB cells. Error bars represent the SD of the mean. Original blots/gels are presented in Supplementary Fig. 1.*ns* non-significant, *p < 0.05, **p < 0.01, ***p < 0.001, ****p < 0.0001.

The immunofluorescent (IF) staining of CORO1C, SV2B, and β-actin in HA-bs cells demonstrated uniform staining patterns (Fig. 5a). CORO1C exhibited nuclear localisation, while SV2B was localised in the cytoplasm. IF staining in DAOY MB cells showed a significant increase in SV2B fluorescence compared to HA-bs cells, indicating higher expression levels (Fig. 5b). CORO1C maintained a similar nuclear localisation in DAOY cells as observed in HA-bs cells. IF staining in U251MG and U87MG GB cells revealed significantly increased fluorescence of CORO1C compared to HA-bs cells, indicating elevated expression (Fig. 5c). The IF staining for SV2B in GB cells was previously reported by our team and revealed elevated SV2B expression^33^. Western blot analysis showed the expression levels of β-actin, CORO1C, and SV2B across HA-bs, U251MG, U87MG, and DAOY cells (Fig. 5d). CORO1C protein expression was significantly upregulated in all GB (U251MG and U87MG) and MB (DAOY) cell lines compared to HA-bs cells (Fig. 5e). SV2B protein expression was significantly increased in U251MG, U87MG, and DAOY cells compared to HA-bs cells (Fig. 5f). β-actin protein expression did not show any significant differences between normal astrocytes and GB or MB cells, serving as a consistent loading control (Fig. 5g).

### *CORO1C* expression is elevated in MB and GB and increases with tumour grade and patient age

**Fig. 6:**
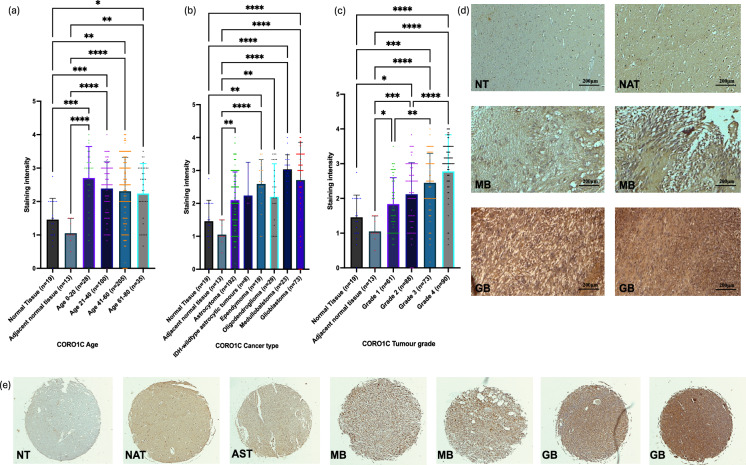
Immunohistochemistry analyses of CORO1C. (**a**) All age groups showed significantly increased levels of CORO1C in comparison to their normal counterparts. The age groups of 0–20 years showed the most significantly increased levels of CORO1C in comparison to normal and normal adjacent tissues controls (Normal Tissue versus Age 0–20, p < 0.0001; Adjacent brain tissue versus Age 0–20, p < 0.0001; Normal Tissue versus Age 21–40, p < 0.0009; Adjacent Brain Tissue versus Age 21–40, p < 0.0001; Normal Tissue versus Age 41–60, p < 0.0019; Adjacent Brain Tissue versus Age 41–60, p < 0.0001; Normal Tissue versus Age 61–80, p < 0.0360; Adjacent Brain Tissue versus Age 61–80, p < 0.0012). (**b**) Significantly higher levels of the CORO1C protein were observed within MB and GB, in comparison to the rest of the malignancies and their normal counterparts (Adjacent Brain Tissue versus Astrocytoma, p < 0.0014; Normal Tissue versus Ependymoma, p < 0.0027; Adjacent Brain Tissue versus Ependymoma, p < 0.0001; Adjacent Brain Tissue versus Oligodendroglioma, p < 0.0037; Normal Tissue versus MB, p < 0.0001; Adjacent Brain Tissue versus MB, p < 0.0001; Normal Tissue versus GB, p < 0.0001; Adjacent Tissue versus GB, p < 0.0001). (**c**) Significant differences between tumour grades 2, 3 and 4 in comparison to normal brain tissue were observed. Grade 1 tumours showed significantly higher expression in comparison to normal adjacent tissue. Significant differences between grades 3 and 4 and grades 1 and 2 (Adjacent Brain Tissue versus Grade 1, p < 0.0460; Normal Tissue versus Grade 2, p < 0.0395; Adjacent Brain Tissue versus Grade 2, p < 0.0009; Normal Tissue versus Grade 3, p < 0.0003; Adjacent Brain Tissue versus Grade 3, p < 0.0001; Normal Tissue versus Grade 4, p < 0.0001; Adjacent Brain Tissue versus Grade 4, p < 0.0001; Grade 1 versus Grade 3, p < 0.0012; Grade 1 versus Grade 4, p < 0.0001; Grade 2 versus Grade 4, p < 0.0001). (**d**) Microscopic images of CORO1C stained tissues sections illustrating the staining intensity within normal brain tissue, tumour adjacent tissue, MB and GB tissues at × 100 magnification. (**e**) Microscopic images of CORO1C stained tissues cores of normal brain tissue, tumour adjacent tissue, and different brain malignancies at × 40 magnification. Results are based on three independent scoring assessments, n = 3. All statistical analyses were conducted using ordinary one-way ANOVA, followed by Dunnett’s multiple comparisons test to determine the significant differences in CORO1C expression. Error bars represent the SD of the mean.*ns* non-significant, *p < 0.05, **p < 0.01, ***p < 0.001, ****p < 0.0001, *NT* normal tissue, *NAT* normal adjacent tissue, *AST* astrocytoma, *MB* medulloblastoma, *GB* glioblastoma.

Immunohistochemical (IHC) analysis of CORO1C was conducted on patient cohorts and normal brain samples, revealing higher protein expression in patients across all age groups compared to normal and adjacent normal brain tissues. Notably, significant differences in protein expression were observed in all age groups, with the highest significance being within the 0–20 years age groups when compared to their normal counterparts (Fig. 6a). Figure 6b demonstrates varying CORO1C expression patterns across different tumour types. The highest significant difference in CORO1C levels was observed between MB and GB in comparison to their normal counterparts. Malignant brain tumours, in general, expressed higher CORO1C levels than normal and adjacent normal tissues, with these differences reaching statistical significance. A significant increase in CORO1C expression was also observed with advancing tumour grade (Fig. 6c). Significant differences in CORO1C levels were observed between all grades, with grade 4 tumours exhibiting much higher CORO1C levels than lower-grade tumours. Microscopic images in Fig. 6d show increased brown staining intensity in high-grade tumours, including MB and GB, illustrating the elevated CORO1C expression. Figure 6e panels display different patient samples at various tumour stages, further emphasising the progression-related increase in CORO1C expression.

### miR-206 and miR-383 mimic transfection downregulates *CORO1C* and *SV2B* and reduces tumorigenic potential in MB cells

**Fig. 7:**
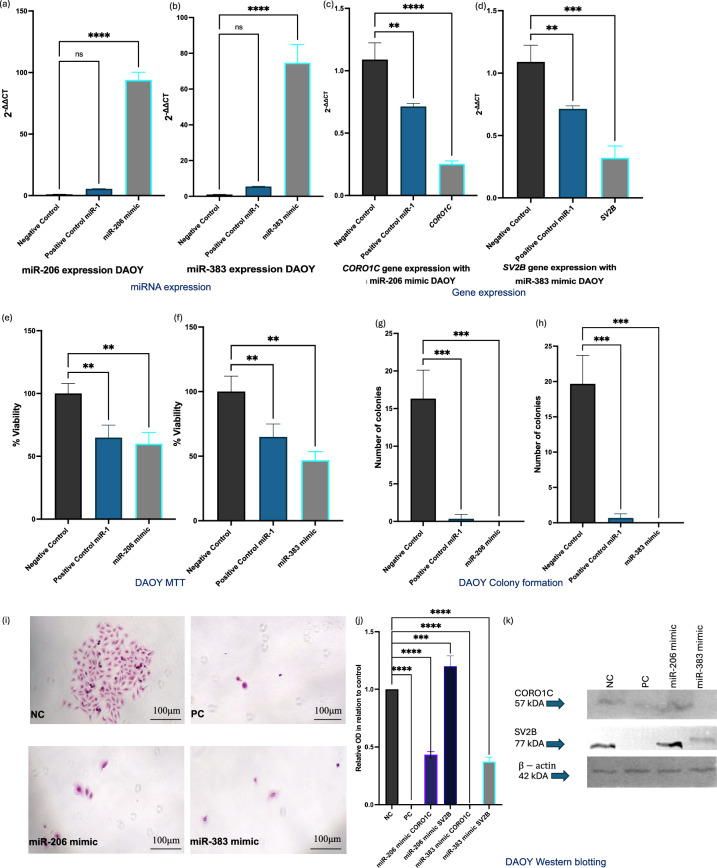
Transient transfections of MB cells. (**a, b**) miR-206 and miR-383 were significantly upregulated after transfections with miR-206 and miR-383 mimics (miR-206, p < 0.0001; miR-383, p < 0.0001). (**c**) *CORO1C* was significantly downregulated post transfection with miR-206 mimic (p < 0.0009). (**d**) *SV2B* was significantly downregulated post transfection with miR-383 mimic (p < 0004). (**e, f**) MB cell viability was significantly decreased post transfection with miR-206 and miR-383 mimics (miR-206, p < 0.0032; miR-383, p < 0.009). (**g, h**) MB cells lost their colony forming abilities post transfections with miR-206 and miR-383 mimics. (**i**) Microscopic images of CF within MB cells post exposure to miR-206 and miR-383 mimics (× 40 magnification). (**j**) Western blotting analysis showed that the protein levels of CORO1C were significantly decreased in comparison to the negative control condition post exposure to miR-206 mimic (p < 0.0001). The protein levels of SV2B were also significantly reduced in comparison to the negative control condition post exposure to miR-383 mimic (p < 0.0009). (**k**) Chemiluminescent images showing the expression of β-actin, CORO1C, and SV2B within negative control miRNA #1, positive control miR-1, and miR-206 and miR-383 mimics. Results are based on three independent experiments, n = 3. All statistical analyses were conducted using ordinary one-way ANOVA, followed by Dunnett’s multiple comparisons test. Error bars represent the SD of the mean. Original blots/gels are presented in Supplementary Fig. 1.* ns* non-significant, *p < 0.05, **p < 0.01, ***p < 0.001, ****p < 0.0001.

Transient transfections of MB cells with miR-206 and miR-383 mimics resulted in significant upregulation of these miRNAs. Figures 7a,b illustrate the increased expression levels of miR-206 and miR-383 following transfection, confirming the effectiveness of the mimics. Following the transfection of MB cells with miR-206 mimics, a significant downregulation of *CORO1C* was observed (Fig. 7c). Similarly, transfection with miR-383 mimics led to a significant reduction in *SV2B* expression (Fig. 7d). Post-transfection with miR-206 and miR-383 mimics, there was a significant decrease in MB cell viability (Figs. 7e-f). MB cells transfected with miR-206 and miR-383 mimics also demonstrated a marked loss of colony-forming abilities (Figs. 7g-h). Microscopic images (Fig. 7i) of colony formation within MB cells post-transfection with miR-206 and miR-383 mimics (at 40 × magnification) visually confirmed the loss of colony-forming ability. Western blotting analysis revealed that the protein levels of CORO1C were significantly decreased following transfection with the miR-206 mimic compared to the negative control condition (Fig. 7j). Similarly, the protein levels of SV2B were significantly reduced after transfection with the miR-383 mimic compared to the negative control condition (Fig. 7j). Chemiluminescent images showed the expression levels of β-actin, CORO1C, and SV2B across different conditions, including the negative control miRNA #1, positive control miR-1, and the miR-206 and miR-383 mimics (Fig. 7k). β-actin was consistently expressed across all samples, confirming equal protein loading. CORO1C expression was visibly decreased in the miR-206 mimic condition, and SV2B expression was reduced in the miR-383 mimic condition. To further validate these findings in a second MB cell line, D425 cells were transfected with miR-206 and miR-383 mimics (see Supplementary Fig. 3 online). Transfection efficiency was confirmed by a significant upregulation of miR-206 and miR-383 expression compared to control condition (see Supplementary Fig. 3a and 3b online). Respectively, miR-206 transfection resulted in a marked downregulation of *CORO1C*, while miR-383 mimic significantly suppressed *SV2B* expression in D425 cells (see Supplementary Fig. 3c and 3d online).

### miR-206 and miR-383 reduce *CORO1C* and *SV2B *expression and inhibit viability and colony formation in GB cells

**Fig. 8:**
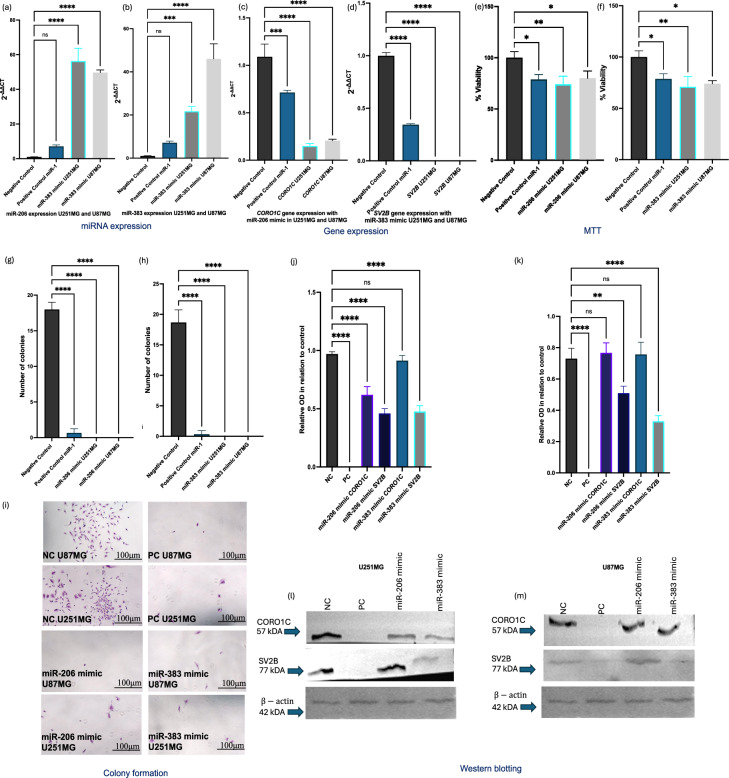
Transient transfections of GB cells. (**a, b**) miR-206 and miR-383 were significantly upregulated after transfections with miR-206 and miR-383 mimics within both GB cell lines (U251MG miR-206, p < 0.0001; U87MG miR-206, p < 0.0034; U251MG and U87MG miR-383, p < 0.0001). (**c**) *CORO1C* was significantly downregulated post transfection with miR-206 mimic in U251MG and U87MG cells (U251MG, p < 0.0001; U87MG, P < 0.0001). (**d**) *SV2B* was significantly downregulated post transfection with miR-383 mimic with both GB cell lines (U251MG, p < 0.0001; U87MG, p < 0.0001). (**e, f**) GB cell viability was significantly decreased post transfection with miR-206 and miR-383 mimics in both, U251MG and U87MG cells (U251MG miR-206, p < 0.0039; U87MG miR-206, p < 0.04; U251MG miR-383, p < 0.002; U87MG miR-383, p < 0.001). (**g, h**) GB cells lost their colony forming abilities post transfections with miR-206 and miR-383 mimics (p < 0.0001). (**i**) Microscopic images of CF within GB cells post exposure to miR-206 and miR-383 mimics (× 40 magnification). (**j**) Western blotting analysis in U251MG showed that the protein levels of CORO1C were significantly decreased in comparison to the negative control condition post exposure to miR-206 mimic (p < 0.0001), whilst the levels of CORO1C were unaffected in the miR-383 mimic condition. The protein levels of SV2B were also significantly reduced in comparison to the negative control condition post exposure to miR-383 mimic (p < 0.0001). Additionally, the levels of SV2B were also downregulated in the miR-206 mimic condition (p < 0.0001). (**k**) Western blotting analysis in U87MG showed that the protein levels of CORO1C were significantly decreased in comparison to the negative control condition post exposure to miR-383 mimic p < 0.0002), whilst the levels of CORO1C were unaffected in the miR-206 mimic condition. The protein levels of SV2B were also significantly reduced in comparison to the negative control condition post exposure to miR-383 mimic(p < 0.0001). Additionally, the levels of SV2B were not affected in the miR-206 mimic condition. (**l, m**) Chemiluminescent images showing the expression of β-actin, CORO1C, and SV2B within U251MG and U87MG cells post exposure to negative control miRNA #1, positive control miR-1, and miR-206 and miR-383 mimics. Results are based on three independent experiments, n = 3. All statistical analyses were conducted using ordinary one-way ANOVA, followed by Dunnett’s multiple comparisons test. Error bars represent the SD of the mean. Original blots/gels are presented in Supplementary Fig. 1**. ***ns* non-significant, *p < 0.05, **p < 0.01, ***p < 0.001, ****p < 0.0001.

Figures 8a,b demonstrated the increased expression levels of miR-206 and miR-383 following transfection of U251MG and U87MG GB cells. Post-transfection with miR-206 mimics, a significant downregulation of *CORO1C* was observed in both GB cell lines (Fig. 8c). Additionally, transfection with miR-383 mimics led to a significant reduction in *SV2B* expression (Figured 8d). The transfection of GB cells with miR-206 and miR-383 mimics also resulted in a significant decrease in cell viability within both cell lines (Figs. 8e-f). Additionally, GB cells exhibited a reduced colony-forming potential post-transfection with these miRNA mimics (Figs. 8g-h). Microscopic images (Fig. 8i) of colony formation in GB cells post-transfection with miR-206 and miR-383 mimics (at 40 × magnification) visually confirmed the loss of colony-forming ability. Western blotting analysis in U251MG cells showed a significant decrease in CORO1C protein levels following transfection with the miR-206 mimic compared to the negative control condition. However, CORO1C levels remained unaffected when transfected with the miR-383 mimic (Fig. 8j). The protein levels of SV2B were significantly reduced post-transfection with the miR-383 mimic compared to the negative control condition. Additionally, SV2B levels were also downregulated in the miR-206 mimic condition (Fig. 8j). In U87MG cells, CORO1C protein levels significantly decreased following transfection with the miR-383 mimic, while CORO1C levels remained unaffected by the miR-206 mimic (Fig. 8k). SV2B protein levels were significantly reduced in the miR-383 mimic condition compared to the negative control. In contrast, SV2B levels were not affected by the miR-206 mimic in U87MG cells (Fig. 8k). Chemiluminescent images displayed the expression levels of β-actin, CORO1C, and SV2B in U251MG and U87MG cells post-transfection with negative control miRNA #1, positive control miR-1, and the miR-206 and miR-383 mimics (Figs. 8l,m). β-actin was consistently expressed across all samples, serving as a loading control. The images revealed the western blot findings, showing decreased CORO1C expression in U251MG with miR-206 and in U87MG with miR-383, as well as reduced SV2B expression in U251MG with both miR-206 and miR-383, and in U87MG with miR-383.

## Discussion

MBs and GBs are the most aggressive brain tumours found in children and adults, respectively. Limited effective treatments face challenges such as tumour recurrence, resistance, and significant side effects^34^. The potential use of miRNAs in therapy is promising, as miRNAs can regulate gene expression involved in tumour growth and resistance^35^. Thus, targeting specific miRNAs could provide a novel, less invasive, and more precise therapeutic approach.

Our small-RNA sequencing results depicted that a total of 328 miRNAs were significantly dysregulated in MB patient samples when compared to normal brain tissue. miR-206 and miR-383 were amongst the most significantly downregulated miRNAs in the patient cohort. MiR-206 is located at cytogenetic band 6p12.2 between the *IL-17* and *PKHD1* genes^36^. This miRNA plays a role is the palatogenesis of various conditions and has a physiological role in muscle development, like other members of the miR-1 family to which it belongs^37^. Previous evidence demonstrated the tumour suppressive implications of miR-206 in various malignancies, such as breast, lung and colorectal tumours^38–40^. A recent study by Zhang et al.^41^ has also shown a downregulated expression profile of miR-206 in MB cells. Specifically, they suggested that the *HOTAIR*-miR-1/miR-206-*YY1* axis could be used as a potential therapeutic target for MB treatment^41^. Additionally, miR-206 was found downregulated in other types of aggressive brain neoplasms, such as GB^42^. Located within intron 3 of the *SGC7* gene, miR-383 expression has been observed to be downregulated in various types of cancer, such as breast and pancreatic cancers, suggesting its tumour suppressive role^43,44^. This miRNA has also been observed downregulated in MB tissues and GB cell lines^45,46^. miR-383 was seen to directly target for *FOXM1*, suggesting that the miR-383/*FOXM1* axis could be a potential therapeutic target for MB^45^. We validated our findings in vitro using more sensitive techniques and further demonstrated the downregulation of miR-206 and miR-383 within MB and GB cell lines. Thus, our findings further strengthened the signatory expression of miR-206 and miR-383 in MB and GB, suggesting their potential clinical application as therapeutic targets for highly malignant brain tumours.

The sRNA-seq findings also revealed that the dysregulated miRNAs were linked to axon guidance, natural killer cell-mediated cytotoxicity, and various cancer-related pathways, emphasising their potential roles in MB pathogenesis and progression. Furthermore, they were involved in processes metal ion binding and nucleic acid binding activities, alongside the regulation of transcription and intracellular signal transduction. Previous research has demonstrated that miR-206 modulates the activity of several oncogenic pathways, influencing cancer progression and cellular behaviour. Key pathways affected by miR-206 include the EGF/EGFR/MAPK pathway and the TGF-β pathway, both promoting cell growth and proliferation^47,48^. Additionally, miR-383 has been involved in several cancer pathways, such as the PI3K/AKT/mTOR pathway and the Wnt/β-catenin pathway^49,50^. These pathways are vital for cell growth, differentiation, and response to external stimuli, indicating that miRNA dysregulation in MB could significantly affect cellular behaviour and contribute to tumour development and maintenance.

We further investigated the potential targets of miR-206 and miR-383 using in silico analysis. The base-pair matching analysis performed revealed that miR-206 was directly targeting *CORO1C*, whilst miR-383 was targeting *SV2B*. miR-206 has several important functions in the brain, particularly related to neurogenesis, synaptic plasticity, and neuroprotection^51^. Its target, *CORO1C*, has been shown to regulate the formation and maintenance of lamellipodia, which are sheet-like cell protrusions involved in cell migration which could directly impact tumour cell migration^52^. As seen in our results, miR-206 was targeting *CORO1C* in an 8mer manner, suggesting a stable binding between the miRNA and the mRNA followed by a complete degradation the target mRNA, hence the neuroprotective roles of miR-206. On the other hand, the expression of miR-383 has been observed significantly higher in the marginal division (MrD) than in the hippocampus of Sprague–Dawley rats, suggesting that miR-383 plays an important role in the learning and memory functions associated with the MrD^53^. Previous research has also demonstrated that miR-383 protects against propofol-induced cognitive impairment by preventing hippocampal neuron apoptosis and the dysregulation of associated signalling pathways^54^.Furthermore, it has been shown that in brain tumours, such as gliomas, *SV2B* expression can influence tumour behaviour by affecting synaptic and neuronal environments^33^.​Thus, we could hypothesise that miR-383 plays a significant role in brain and synaptic functions, hence its involvement in learning and its direct *SV2B* gene target. As seen in our results, miR-383 targeted *SV2B* in an 8mer manner, suggesting the strong interaction between the miRNA and mRNA, leading to complete degradation of the target mRNA.

Further transcriptomics analysis of the MB patient samples identified 1013 dysregulated genes, with *CORO1C* being amongst the upregulated genes in MB patients, suggesting its oncogenic capacity. *CORO1C* has been demonstrated to have a significant role in migration and proliferation by influencing actin rearrangement and cofilin dynamics^52^. To the best of our knowledge *CORO1C* has not been associated with MB previously. The association between miR-206 and *CORO1C* has been demonstrated in non-small lung cell carcinomas and triple-negative breast cancer, where deletion or inhibition of *CORO1C* rescued the expression levels of miR-206 and led to supressed metastasis^39^. Our team also showed that miR-206 acted as a potential target for *CORO1C* in paediatric and adult GB^42^. Zhou et al.^55^ suggested that miR-206 could potentially influence the β-catenin growth-dependent mechanism within the Wnt/β-catenin signalling pathway via targeting *CORO1C*^55^. Nevertheless, to the best of our knowledge the link between miR-206 and *CORO1C* in MB has not been previously demonstrated. Factors related to brain development and plasticity during childhood might explain why *CORO1C* levels are elevated in MB paediatric patients. We further validated the upregulation of *CORO1C* in MB and GB cells lines, suggesting that its overexpression could enhance the early identification of paediatric and adult brain tumours and serve as therapeutic targets for their management.

Our transcriptomics analysis also showed that *SV2B* was among the upregulated genes. *SV2B* plays a critical role in the storage and release of neurotransmitters upon neuronal excitation^56^. We have previously demonstrated that the SV2B protein is highly expressed within GB tissues and those patients who exhibited high SV2B levels had shorter overall survival^33^. Given SV2B’s role in synaptically active regions and its involvement in glioma progression, its expression and function might have implications in paediatric MB. Since *SV2B* is expressed in the cerebellum, where MBs commonly arise, it could play a role in the tumour’s biology^57^. SV2B’s function in neurotransmitter release might influence the tumour microenvironment in MB, similar to its effects in gliomas^33^. We further validated the expression of *SV2B* in MB and GB cells and we identified that significantly overexpressed *SV2B* mRNA levels, suggesting that *SV2B* could also serve as a prognostic marker or therapeutic target in MB and GB patients. To the best of our knowledge there is no existing evidence regarding the association between miR-383 and *SV2B*. While SV2B’s specific role in paediatric MB requires further investigation, its established functions and effects in GB provide a basis for exploring its impact on this type of childhood brain cancer.

The dysregulated genes identified in MB were primarily involved in the regulation of the cell cycle, synaptic vesicle cycle, axon guidance, and miRNAs in cancer, highlighting their crucial role in cellular proliferation, communication, and structural integrity, all of which are vital in the context of MB development and progression and correlate the functions of the selected oncogenes, *CORO1C* and *SV2B.* Our in silico analysis revealed distinct prognostic associations for *SV2B* and *CORO1C* in MB and GB. High *SV2B* expression was significantly associated with poorer overall survival in both tumour types, supporting its role as a potential negative prognostic marker and suggesting a contribution to tumour aggressiveness. In contrast, while elevated *CORO1C* expression was significantly linked to reduced survival in MB, it showed a non-significant trend toward improved survival in GB, indicating a possible context-dependent function. These findings highlight the importance of tumour-specific biology in shaping the prognostic impact of these genes and underscore the need for further mechanistic studies to clarify how *SV2B* and *CORO1C* contribute to disease progression in distinct brain tumour subtypes.

Further protein expression analysis via western blotting, IF and IHC also indicated that CORO1C and SV2B were overexpressed in MB and GB cells and tissues, respectively. When tested ex vivo*,* increased expression of CORO1C was observed. The strong significance between the MB and control groups observed through IHC suggested that CORO1C possessed oncogenic properties and that it could serve as a potential therapeutic target. Our IHC findings revealed that CORO1C expression increases with tumour grade, suggesting that highly metabolically active malignancies, such as MB and GB, might require increased expression of this protein for proliferation and metastasis. Our results showed significantly high CORO1C expression in patients aged 0–20 compared to their normal counterparts, indicating its prognostic relevance in paediatric brain malignancies, such as MB. Moreover, the elevated levels of CORO1C in highly malignant paediatric brain malignancies could be associated with its role in influencing cell migration and proliferation. The significant overexpression of CORO1C in the 61–80 age group also suggested its high expression in adult brain malignancies, such as GB. We have also previously reported elevated levels of SV2B in MB and GB patients^33^. The elevated expression levels of CORO1C and SV2B suggested that they could serve as putative prognostic biomarkers, enhancing their clinical relevance in combating highly malignant brain tumours like MB and GB.

Our study investigated the effects of transient transfections of MB and GB cells with miR-206 and miR-383 mimics. Firstly, we observed that the transfections with miR-206 and miR-383 mimics resulted in significant upregulation of these miRNAs, suggesting their utilisation as potential therapeutics against MB and GB. Furthermore, we observed that *CORO1C* was significantly downregulated post-transfection with the miR-206 mimic, suggesting the direct effects of miR-206 upon *CORO1C*. Downregulation of the gene could impair the metastatic potential of MB and GB cells, thus rescuing the malignant phenotype of these neoplasms. Similarly, *SV2B*, was significantly downregulated following transfection with the miR-383 mimic. The reduction in *SV2B* levels suggested a possible decrease in the aggressive characteristics of MB and GB cells, given its association with malignant progression.

The impact of miR-206 and miR-383 mimics on MB and GB cell viability and colony-forming abilities revealed a significantly decreased MB and GB cell viability, highlighting their potential as therapeutic agents in reducing tumour cell survival. Moreover, the loss of colony-forming abilities in these cells post-transfection further supports the notion that these miRNAs can inhibit tumorigenicity. This effect is crucial, as the ability to form colonies is a hallmark of cancer cell proliferation and survival. Taken together, these findings provided significant insights into the potential therapeutic applications of these microRNAs in MB and GB treatment.

Notably, our findings were observed in well-characterised GB and MB cell lines representing the most common and aggressive subtypes. U87MG models the classical GB subtype with *PTEN* mutations and PI3K/Akt activation, although it lacks typical astrocytic markers^58^. This subtype is challenging to treat due to the activation of the PI3K/Akt/mTOR pathway, which promotes cell survival and proliferation, leading to resistance against therapies like temozolomide (TMZ) and EGFR inhibitors^59^. U251MG reflects the mesenchymal subtype with invasive growth, *PTEN* and *p53* mutations, and GFAP expression. The mesenchymal subtype is particularly difficult to manage because of its high invasiveness and resistance to radiotherapy, attributed to its aggressive phenotype^60^. In MB, DAOY cells represent the Sonic Hedgehog (SHH) subtype, and D425 cells model the aggressive Group 3 subtype with high *MYC* expression^61^. However, fewer than half of established MB cell lines have been molecularly subgrouped, with Group 3 and SHH overrepresented, and WNT and Group 4 remaining less studied^61^. To enhance clinical relevance, future in vitro models must incorporate a broader range of well-subtyped cell lines alongside physiologically representative culture systems^61^. In addition, in the current study the molecular subtype information of our patient samples was not available in the biobanks’ directory due to the fact that molecular subtyping was not yet established at the time of collection. While our axes, miR-206/*CORO1C* and miR-383/*SV2B* were validated across GB and MB cell lines, most functional assays were conducted in two GB cell lines and a single MB cell line (DAOY). Exceptions include transfection experiments which were also validated in D425 cell line. These limitations highlight the need for broader validation across more diverse models.

Our findings revealed that the miR-206/*CORO1C* and miR-383/*SV2B* axes are active in both MB and GB, suggesting a potential overlap in the molecular pathways driving these malignancies. *CORO1C* has been associated with enhanced cell migration and invasion through its involvement in the PI3K/AKT and Wnt/β-catenin signalling pathways^62^^.^^63^. As aforementioned, these pathways are frequently dysregulated in both MB and GB and are closely linked to tumour progression and resistance to therapy. In MB, particularly within the SHH subgroup, dysregulation of the SHH pathway has been linked to impaired synaptic signalling and neuronal differentiation, where *SV2B* may contribute by altering vesicle trafficking and neurotransmitter release^64^. In GB, tumour cells form glutamatergic synapses with neurons, and *SV2B* may support this pathological communication by facilitating synaptic vesicle fusion and excitatory signalling that drives tumour growth^65^. Supporting our findings, the observed overlap in synaptic and signalling pathway alterations highlights the role of miR-206 and miR-383 in driving common oncogenic mechanisms across MB and GB.

To our knowledge, this is the first study to highlight the clinical significance of the miR-206/*CORO1C* and miR-383/*SV2B* axes as potential therapeutic targets for paediatric MB and adult GB. Notably, while these axes are present in both malignancies, they seem to operate independently and do not synergise. This finding opens new avenues for shared targeted treatment strategies in children and adults diagnosed with aggressive brain malignancies.

## Methods

### Patients’ samples

For the sRNA sequencing (sRNA-seq) and bulk RNA-seq, resected brain tumours from children diagnosed with MB (n = 9) according to the 2007 WHO criteria were studied^66^. As control, sample was obtained from deceased male child who underwent autopsy and did not exhibit any brain distortion. The patient cohort comprised 4 males and 5 females, aged between 4.02 and 12.05 years, whilst the age for the non-malignant sample was 4 years. For the immunohistochemistry experiments, the slides screened included in total of 376 biopsy cores of malignant and adjacent control tissue (GL2082, GL2083c, GL803c, GL631). Specifically, we tested astrocytoma (n = 192) aged between 19–70 years, IDH-wild type astrocytic tumours (n = 8) aged between 29–66 years, ependymoma (n = 19) agreed between 26–81 years, oligodendroglioma (n = 29) aged between 30–67 years, MB (n = 23), aged between 4–47 years, GB (n = 73) aged between 9–80 years, normal tissues (n = 19) aged between 20–72 years and adjacent normal tissue (n = 13) aged between 30–67 years. The patients’ cohort comprised 213 males and 163 females, whilst the median age for the control cohort was 41 years. All samples were snap-frozen during resection and stored at -80 °C until use. This study was approved by the University of Hertfordshire Ethics Committee (Protocol No. aLMS/PGR/UH/05146(1)), and informed consent was obtained from the parents of all participating children. All methods were performed in accordance with the relevant guidelines and regulations.

### sRNA-seq

In brief, RNA, including miRNA, was extracted following the Trizol method described by Braoudaki et al.^67^. The concentration of each sample was measured with a Nanodrop bioanalyzer (Thermo, model: Nanodrop2000), and RNA integrity was assessed using the Agilent Bioanalyzer 2100, with only samples having RIN numbers above seven qualifying for sRNA-seq at Biomarker Technologies (Germany). Small RNA libraries were prepared as previously described by Mustafov et al.^46^, using the NEBNext® Ultra™ Illumina kit, involving adapter ligation, reverse transcription, PCR amplification, and size selection. Libraries were sequenced on an Illumina HiSeq platform, and raw data was processed to remove low-quality reads and select for lengths between 18–30 nucleotides. Clean reads were aligned against databases to filter out non-coding RNAs and identify miRNAs. Differential expression analysis compared miRNA levels between the control and patient groups, and miRNA target genes were annotated using various databases, including NCBI, Pfam, KOG/COG, Swiss-Prot, KEGG Ortholog, and Gene Ontology. The small-RNA sequencing data was uploaded on Mendeley Data DOI: 10.17632/yrryf4btst.1.

### In silico analysis

A comprehensive miRSystem analysis was employed to identify the gene targets of highly dysregulated selected miRNAs identified through the sRNA-seq analysis (http://mirsystem.cgm.ntu.edu.tw). The Software for Statistical Folding of Nucleic Acids and Studies of Regulatory RNAs (Sfold) was then utilised to provide the probable binding sites of selected miRNAs to their gene targets. Sfold predicted the probable RNA secondary structures through structure ensemble sampling and centroid predictions, with an emphasis on assessing RNA target accessibility (https://sfold.wadsworth.org/cgi-bin/index.pl). Survival analysis for *CORO1C* and *SV2B* was performed using public datasets. For MB, the Cavalli dataset (n = 612) was analysed via the R2 Genomics Platform (https://hgserver1.amc.nl), using gene-specific expression cutoffs (1167.9 for *SV2B*, 1880.9 for *CORO1C*). For GB, the TCGA GB dataset (n = 162) was analysed using GEPIA (http://gepia.cancer-pku.cn), with median expression cutoffs. Kaplan–Meier curves and log-rank tests were used to assess significance, reporting *p*-values and hazard ratios.

### Transcriptomics

RNA previously extracted, as described by Braoudaki et al.^67^, was used. Bulk RNA sequencing was performed at Biomarker Technologies (Germany). The concentration of extracted nucleic acid was measured using the Nanodrop2000 (Thermo, model: Nanodrop2000), and the integrity was assessed using the Agilent 2100 and LabChip GX (Perkin Elmer, model: LabChip GX). The VAHTS Universal V8 RNA-seq Library Prep Kit for Illumina NR605 was employed to construct the mRNA and strand-specific mRNA libraries, following the protocol provided by Vazyme. The prepared libraries were sequenced on the Illumina Novaseq X platform (Illumina, San Diego, CA), and sequencing reads were generated. The mRNA sequencing data was uploaded on Mendeley Data DOI: 10.17632/yrryf4btst.1.

### Cell culture

Two commercially available human GB cell lines, U251MG (Catalogue number: 09063001, human GB cell line used at passages 5–20, Sigma-AldrichTM, Dorset, UK) and U87MG (Catalogue number: ATCC HTB-14, human GB cell line used at passages 5–20, ATCC, Manassas, VA, USA), and MB cell lines, DAOY (Catalogue number: HTB-186, human MB cell line used at passages 1–15, ATCC, Manassas, VA, USA), and D425 (Catalogue number: SCC290, human MB cell line sued at passages 1–15, Sigma-AldrichTM, Dorset, UK) were cultured in Minimum Essential Medium (MEM; Gibco™, Bleiswijk, NL) supplemented with 10% fetal bovine serum (FBS; Gibco™, Bleiswijk, NL) and 1% penicillin–streptomycin (Gibco™, Bleiswijk, NL) to promote growth. Human astrocyte cells isolated from the brain stem (HA-bs) were used as a control cell line (Catalogue number: SCI-1200, human astrocyte cell line used at passages 2–5, CliniSciences, UK). The cells were grown in an astrocyte medium supplemented with FBS, penicillin–streptomycin, and astrocyte growth supplements (CliniSciences, UK). All cell lines were maintained at 37 °C in a 5% CO_2_ incubator.

### Reverse transcription quantitative real-time polymerase chain reaction assays

Total RNA isolation from HA-bs, U251MG, U87MG, and DAOY cells was performed as described by Braoudaki et al.^67^. In brief, total RNA and miRNAs were extracted following the Trizol reagent protocol and mirVana isolation kit (ThermoFisher, Vilnius, Lithuania), respectively. Following sample treatment with RNase-free DNase (Qiagen, Hilden, Germany), the quantity and quality of the sample were assessed by using Nanodrop (Nanodrop ND1000 Spectrophotometer, Hampton, USA). cDNA synthesis reactions were performed by a Thermal cycler (Eppendorf, Mastercycler nexus gradient) using a High-Capacity cDNA Reverse Transcription Kit (Applied Biosystems ThermoFisher, Pleasanton, CA). RT-qPCR experiments were performed by using QuantStudio™ Real-Time PCR (Quant Studio 7 flex, Applied Biosystems, Massachusetts, US). Expression analysis in different samples was performed by using specific primers for each gene and miRNAs. 2^-∆∆Ct^ values of fold expression were used to compare the relative differences of expression.

### Western blotting

Extraction of total protein from HA-bs, U251MG, U87MG, and DAOY cell lines was performed using a RIPA buffer and protease inhibitor cocktail (Applied Biosystems ThermoFisher, Pleasanton, CA). Then, electrophoretic separation via 10% SDS polyacrylamide gels under reducing conditions and semi-dry proteins electro-transfer to polyvinylidene difluoride (PVDF) membranes was performed (Amersham Biosciences, Upsala, Sweden). After blocking with 5% bovine serum albumin (BSA) in TBST solution for 1 h at room temperature (RT), membranes were washed three times with TBST-0.1% Tween-20. Following this, the PVDF membranes were incubated with TBST and the appropriate primary antibody antisera against CORO1C (dilution 1:500), SV2B (dilution 1:500), and β-actin (dilution 1:1000) overnight at 4 °C (ProteinTech, Manchester, UK). Next, membranes were washed three times with TBST and incubated in the dark with an appropriate HRP-conjugated secondary antibody for 1 h. Band quantification was performed with the iBright Imaging Systems iBright Analysis Software (Applied Biosystems ThermoFisher, Pleasanton, CA) via chemiluminescent detection and visualisation of proteins. β-actin was used as a housekeeping protein. All antibodies were purchased by ProteinTech, Manchester, UK.

### Immunofluorescence

Immunofluorescence (IF) was carried out as previously described by Mustafov et al.^33^. HA-bs, U251MG, U87MG, and DAOY cells were incubated with the CORO1C primary antibody (1:500 dilution), whilst HA-bs and DAOY were also incubated the SV2B primary antibody (1:500 dilution) (ProteinTech, Manchester, UK). For secondary antibody alone controls, cells were incubated without primary antibody. All cells were then incubated with the secondary antibody Alexa Fluor 488 Phalloidin (Applied Biosystems ThermoFisher, Pleasanton, CA) at a 1:500 dilution. Fluorescent images were obtained using a Zeiss Axioimager M2 microscope equipped with an Axiocam 503 imaging device and Zeiss ZEN software (Zeiss Microscopy, 40 × magnification).

### Immunohistochemistry

Paraffin-embedded brain tumour tissue array slides (US Biomax Inc., London, UK) were acquired following Health Insurance Portability and Accountability Act (HIPAA) approved protocols to ensure ethical standards. The protocol used was previously described by Mustafov et al.^33^. After deparaffinization, rehydration, antigen retrieval, H_2_O_2_ treatment, and blocking, the slides were incubated overnight at 4 °C with the CORO1C primary antibody (ProteinTech, Manchester, UK). Following this overnight incubation, the slides were incubated with a secondary anti-rabbit antibody from the Zytochem Plus HRP-DAB Kit (HRP008DAB-RB, Zytomed Systems, UK) for 1 h Streptavidin-HRP conjugate was then added, followed by a 45-min incubation. Staining and counterstaining were performed with DAB and haematoxylin, respectively. Immunoreactivity was assessed by three independent observers using a light microscope (Zeiss Microscopy, Oberkochen, Germany), scoring the extent of brown staining based on the percentage of positively stained cells.

### Transient transfections

U251MG, U87MG, DAOY, and D425 cell lines were transfected with negative control miRNA #1, positive control miR-1, and miR-206 and miR-383 mimics (Applied Biosystems ThermoFisher, Pleasanton, CA) using the Lipofectamine RNAiMAX reagent (Applied Biosystems ThermoFisher, Pleasanton, CA) as per the manufacturer’s guidelines. All transfections were carried out in 6-well plates using 2.5 × 10^5^ cells/well. After reaching a confluence of between 60–80%, the cells were transfected with the siRNAs at final concertation 30 pmol. This was achieved by performing the following dilutions: 9μL of Lipofectamine RNAiMAX mixed 150μL of Opti-MEM medium (Applied Biosystems ThermoFisher, Pleasanton, CA); and 18μL of siRNA stock solution (5 nmol) mixed with 150μL of Opti-MEM medium. After the dilution was made, both mixtures were added together and incubated for 10 min at RT. Total RNA, miRNA, and protein from the transfected cells were extracted for analysis of miR-206, miR-383, *CORO1C*, *SV2B*, CORO1C, and SV2B expression, respectively. The concentrations of the siRNAs used were 10 nM after mixing them with the transfection reagent (Lipofectamine RNAiMAX) as per the manufacturer’s instructions. The cells were harvested for RNA, miRNA, and protein extraction after 48 h for RT-qPCR and western blot analysis as described above.

### Cell viability assay

The cell viability of transfected U251MG, U87MG, and DAOY cells was assessed as previously described by Mustafov et al.^68^. GB and MB cells were seeded in 96-well plates at a cell density of 1.5 × 10^4^ per well and subsequently transfected with negative control miRNA #1, positive control miR-1, and miR-206 and miR-383 mimics (Applied Biosystems ThermoFisher, Pleasanton, CA) using the Lipofectamine RNAiMAX reagent (Applied Biosystems ThermoFisher, Pleasanton, CA) as per the manufacturer’s guidelines. Following a 48 h incubation with the siRNAs, 5 mg/mL 3-(4,5-dimethylthiazol-2-yl)-2,5-diphenyltetrazolium bromide (MTT) dye (GibcoTM, Bleiswijk, NL) was added in each well and plates were incubated and subsequently read using a CLARIOstar microplate reader (BMG Labtech, Aylesbury, UK).

### Colony forming assay

To assess the ability of U251MG, U87MG, and DAOY cells to form colonies, cells were seeded at a density of 500 cells/well on 12-well plates and transfected with negative control miRNA #1, positive control miR-1, and miR-206 and miR-383 mimics (Applied Biosystems ThermoFisher, Pleasanton, CA) using the Lipofectamine RNAiMAX reagent (Applied Biosystems ThermoFisher, Pleasanton, CA) as per the manufacturer’s guidelines. Post five days of incubation at 37 °C, the colonies were washed with phosphate-buffered saline PBS, fixed with 4% paraformaldehyde (PFA) (Sigma-AldrichTM, Dorset, UK) for 25 min, and stained with 0.1% crystal violet (Sigma-AldrichTM, Dorset, UK). Colonies with more than 30 cells were microscopically photographed and quantitatively assessed under a light microscope (Olympus Life Science Solutions, Stansted, UK).

### Statistics

Statistical analysis and graphical representations were performed using GraphPad Prism 9.4.1 software (GraphPad Software, San Diego, USA). For comparisons involving three or more groups, one-way ANOVA followed by Dunnett’s multiple comparisons test was used. A p-value of < 0.05 was considered statistically significant. For sRNA-seq and transcriptomics, p-values were adjusted using the Benjamini and Hochberg method to control the false discovery rate. miRNAs or genes with an adjusted p-value < 0.05 were considered differentially expressed. A q-value < 0.005 and |log2(fold change)|≥ 1 were set as thresholds for significant differential expression. The log2 fold change > 1 threshold was selected to identify miRNAs and genes with meaningful expression changes that may still be biologically relevant, even if they do not meet the higher fold change threshold of > 2.

## Supplementary Information

Supplementary Information 1.

Supplementary Information 2.

Supplementary Information 3.

Supplementary Information 4.

## Data Availability

The small-RNA sequencing data and the mRNA sequencing data were uploaded on Mendeley Data DOI: 10.17632/yrryf4btst.1. The embargo on the datasets has now been lifted, and the data have been made freely available in accordance with the journal’s data availability policies at the time of resubmission.
