# Supplementary figures and images for: New microRNA-based therapies reveal common targets in paediatric medulloblastoma and adult glioblastoma
Source: Sci Rep. 2025 Jul 2;15:23044. doi: 10.1038/s41598-025-05517-9 (PMC12218976; doi:10.1038/s41598-025-05517-9)

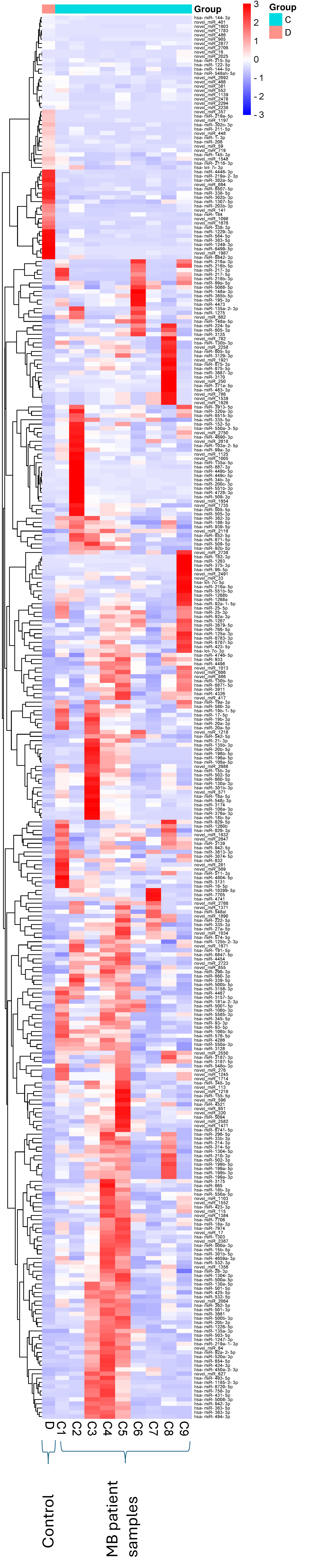

Supplement: Supplementary file 1 — Supplementary Information 1. [file 41598_2025_5517_MOESM1_ESM.pdf]
