# Supplementary material for: New microRNA-based therapies reveal common targets in paediatric medulloblastoma and adult glioblastoma
Source: Sci Rep. 2025 Jul 2;15:23044. doi: 10.1038/s41598-025-05517-9 (PMC12218976; doi:10.1038/s41598-025-05517-9)
Supplement: Supplementary file 3 — Supplementary Information 3. [file 41598_2025_5517_MOESM3_ESM.pdf]

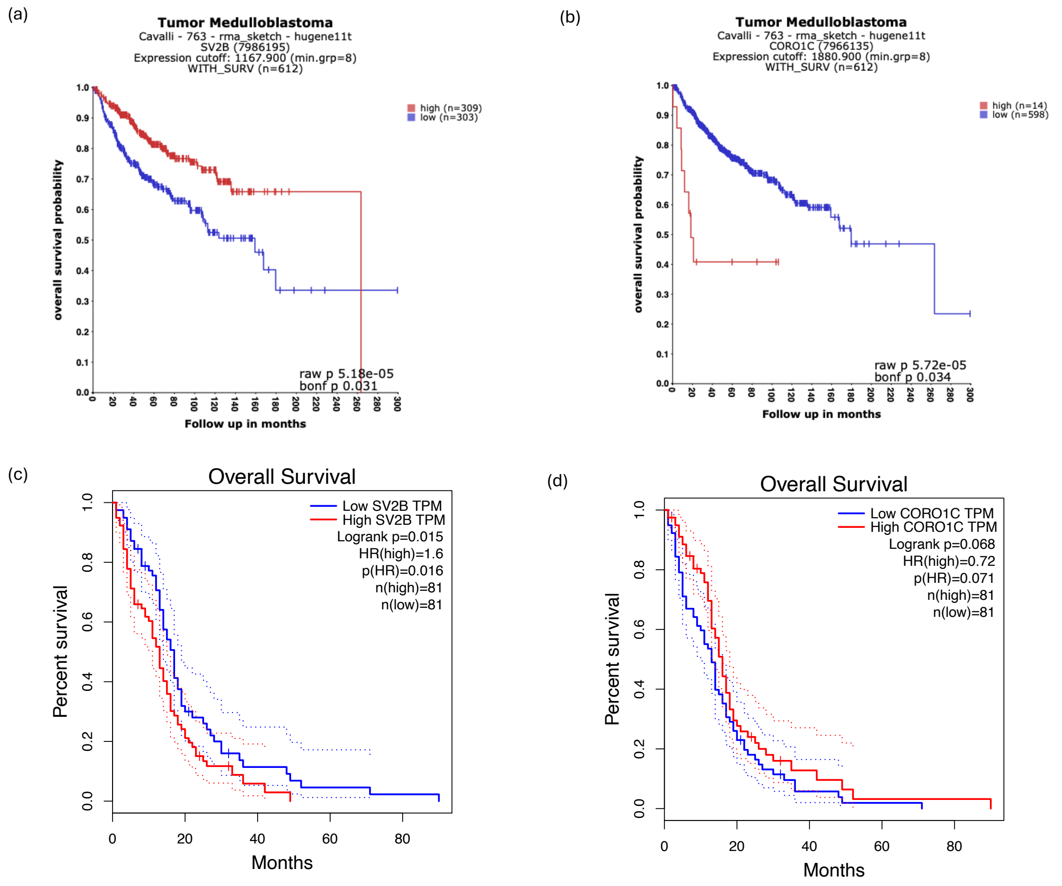

Supplementary Figure 2. Kaplan–Meier overall survival analysis of *SV2B* and *CORO1C* expression in MB and GB patient cohorts. (a) High *SV2B* expression was significantly associated with reduced overall survival in MB patients from the Cavalli dataset (n = 612), using an expression cutoff of 1167.9 (raw p = 5.18e-05; Bonferroni-adjusted p = 0.031). **(b)** Similarly, high *CORO1C* expression correlated with poorer overall survival in the same cohort (expression cutoff = 1880.9; raw p = 5.72e-05; Bonferroni-adjusted p = 0.034). **(c)** In GB patients (n = 162) from the TCGA dataset accessed via GEPIA, high *SV2B* expression was significantly associated with worse overall survival (HR = 1.6; p = 0.015), based on the median expression cutoff. **(d)** High *CORO1C* expression in GB showed a non-significant trend toward improved survival (HR = 0.72; p = 0.068), also using the median cutoff. Survival differences were assessed using the log-rank test.
