# Supplementary material for: New microRNA-based therapies reveal common targets in paediatric medulloblastoma and adult glioblastoma
Source: Sci Rep. 2025 Jul 2;15:23044. doi: 10.1038/s41598-025-05517-9 (PMC12218976; doi:10.1038/s41598-025-05517-9)
Supplement: Supplementary file 4 — Supplementary Information 4. [file 41598_2025_5517_MOESM4_ESM.pdf]

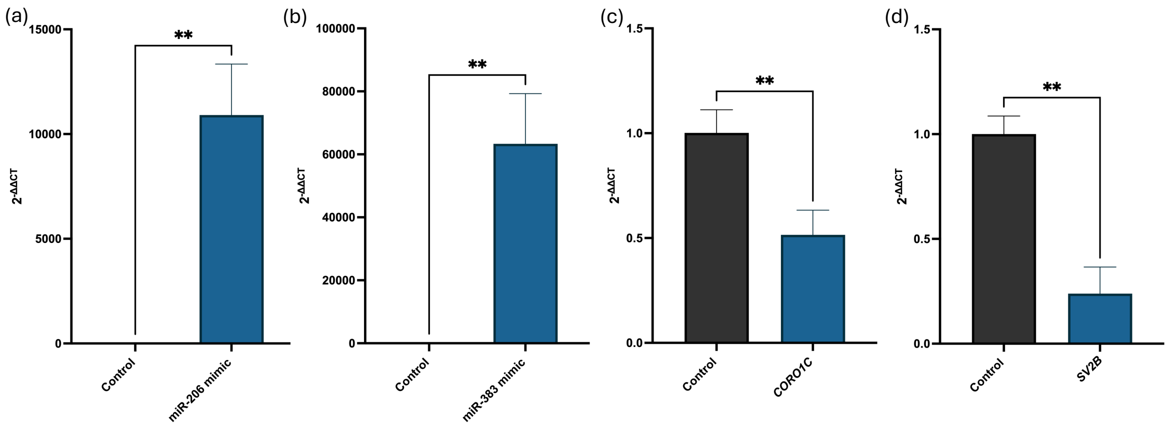

Supplementary Figure 3. Validation of miR-206 and miR-383 function in D425 MB cells. (a, b) Transfection with miR-206 and miR-383 mimics led to a significant increase in their expression levels compared to control (p<0.0001), confirming transfection efficiency. **(c)** miR-206 overexpression significantly downregulated *CORO1C* expression (p<0.01). **(d)** miR-383 overexpression significantly reduced *SV2B* expression (p<0.001). Results are based on three independent experiments, n=3. Statistical analyses were conducted using unpaired t-tests to determine significant differences in expression. Error bars represent the standard deviation (SD) of the mean. **Legends**: ns: non-significant, *p<0.05, **p<0.01, ***p<0.001, ****p<0.0001.
